# Unveiling anticancer, antimicrobial, and antioxidant activities of novel synthesized bimetallic boron oxide–zinc oxide nanoparticles†

**DOI:** 10.1039/d3ra03413e

**Published:** 2023-07-12

**Authors:** Amr H. Hashem, Samar H. Rizk, Mostafa A. Abdel-Maksoud, Wahidah H. Al-Qahtani, Hamada AbdElgawad, Gharieb S. El-Sayyad

**Affiliations:** a Botany and Microbiology Department, Faculty of Science, Al-Azhar University Nasr City Cairo 11884 Egypt amr.hosny86@azhar.edu.eg; b Department of Biochemistry, Faculty of Pharmacy, Ahram Canadian University Sixth of October City Giza Egypt; c Department of Biochemistry, Faculty of Pharmacy, Galala University New Galala City Suez Egypt; d Botany and Microbiology Department, College of Science, King Saud University P.O. Box 2455 Riyadh 11451 Saudi Arabia; e Department of Food Sciences & Nutrition, College of Food and Agricultural Sciences, King Saud University P.O. Box 270677 Riyadh 11352 Saudi Arabia; f Laboratory for Molecular Plant Physiology and Biotechnology, Department of Biology, University of Antwerp 2020 Antwerp Belgium; g Microbiology and Immunology Department, Faculty of Pharmacy, Ahram Canadian University Sixth of October City Giza Egypt; h Microbiology and Immunology Department, Faculty of Pharmacy, Galala University New Galala City Suez Egypt Gharieb.Elsayyad@gu.edu.eg; i Drug Microbiology Lab, Drug Radiation Research Department, National Center for Radiation Research and Technology (NCRRT), Egyptian Atomic Energy Authority (EAEA) Cairo Egypt

## Abstract

Bimetallic nanoparticles have received much attention recently due to their multifunctional applications, and synergistic potential at low concentrations. In the current study, bimetallic boron oxide–zinc oxide nanoparticles (B_2_O_3_–ZnO NPs) were synthesized by an eco-friendly, and cost-effective method through the utilization of gum arabic in the presence of gamma irradiation. Characterization of the synthesized bimetallic B_2_O_3_–ZnO NPs revealed the successful synthesis of bimetallic NPs on the nano-scale, and good distribution, in addition to formation of a stable colloidal nano-solution. Furthermore, the bimetallic B_2_O_3_–ZnO NPs were assessed for anticancer, antimicrobial and antioxidant activities. The evaluation of the cytotoxicity of bimetallic B_2_O_3_–ZnO NPs on Vero and Wi38 normal cell lines illustrated that bimetallic B_2_O_3_–ZnO NPs are safe in use where IC_50_ was 384.5 and 569.2 μg ml^−1^, respectively. The bimetallic B_2_O_3_–ZnO NPs had anticancer activity against Caco 2 where IC_50_ was 80.1 μg ml^−1^. Furthermore, B_2_O_3_–ZnO NPs exhibited promising antibacterial activity against *E. coli*, *P. aeruginosa*, *B. subtilis* and *S. aureus*, where MICs were 125, 62.5, 125 and 62.5 μg ml^−1^ respectively. Likewise, B_2_O_3_–ZnO NPs had potential antifungal activity against *C. albicans* as unicellular fungi (MIC was 62.5 μg ml^−1^). Moreover, B_2_O_3_–ZnO NPs displayed antioxidant activity (IC_50_ was 102.6 μg ml^−1^). In conclusion, novel bimetallic B_2_O_3_–ZnO NPs were successfully synthesized using gum arabic under gamma radiation, where they displayed anticancer, antimicrobial and antioxidant activities.

## Abstract 2

In the current study, bimetallic boron oxide–zinc oxide nanoparticles were synthesized by an eco-friendly and cost-effective method through the utilization of gum arabic and gamma irradiation for elevating the antimicrobial and antioxidant activities.

## Graphical abstract

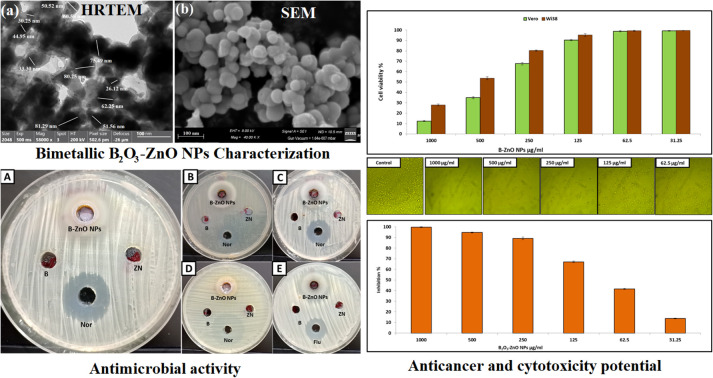

## Introduction

With the rapid spread of infectious diseases that are resistant to treatment, microbial contagious epidemics are becoming among the leading causes of morbidity and mortality. Drug resistance can also develop due to bacterial changes and modifications to efflux pathways that restrict the passage of medication.^1^ Microbes produce enzymes that can change, passivate, or worsen the antibiotic action, which explains how resistance to antibiotics develops. Notably, it has been anticipated that by 2050, drug-resistant diseases will cause over 10 million deaths more than cancer if the trend keeps moving forward at the current rate.^2^ According to predictions, one in five people will develop cancer at some point in their lives, and one in ten will pass away from it. Globally, there were almost 19.3 million new instances of cancer in 2020 alone, and 10 million people died from the disease.^3^ According to Jamalipour *et al.*,^4^ cancer can lead to unchecked cell proliferation through the lymphatic and circulatory systems that has a high potential to invade and spread to other cells and tissues of the body. One of the most important differences between cancer cells and somatic cells is their potential for multiplication and invasion of numerous physiological locations.^5^ Now the most widely used cancer therapies are surgery, radiation therapy, immunotherapy, hormone therapy, and chemotherapy.^6^ However, recently, many researchers have been concentrating on nanomaterials and all-natural cancer treatments.^7–9^

Over the last ten years, experts in a number of fields have been interested in the science of nanotechnology. In this context, the production and design of extremely small particles with a diameter ranging from 1 to 100 nm and is known as nanoparticles (NPs).^10^ Recent investigations in the medicinal, agricultural, energy, environmental health, and industrial domains all heavily include nanotechnology.^11–13^

As the name implies, bimetallic nanoparticles are made up of two distinct metal components.^14^ They have improved catalytic characteristics over monometallic nanoparticles, which is their main benefit over them.^15^ Bi-metallization both enhances the original single-metal catalyst's properties and adds a new one. The second metal's inclusion enables the catalyst's biological activity, selectivity, and stability to be controlled in some processes.^16^ Bimetallic NPs have received a great interest in the scientific and technical domains over the past 10 years due to their distinctive optical, electrical, magnetic, and catalytic capabilities, which are often markedly different from those of their monometallic counterparts. Bimetallic NPs can have a variety of morphologies and structures and are prepared by mixing two different kinds of metal nanoparticles.^17^

Chemical processes are commonly used to synthesize nanomaterials. Despite the treatments' great effectiveness, they are costly and harmful to the environment.^18,19^ Recently, biological approaches for the production of nano-metals have been used, including those involving plants, algae, fungus, biological macromolecules, and bacteria.^7,20–27^ ZnO nanoparticles are widely used in a variety of fields, such as medicine, the environment, and the pharmaceutical industry.

Herein, this study aims to (1) synthesize bimetallic boron oxide–zinc oxide nanoparticles through effective and ecofriendly method, (2) characterize these bimetallic nanoparticles using UV-Vis, HRTEM, SEM, XRD and zeta potential, (3) assess their antimicrobial, antioxidant as well as anticancer activities.

## Materials and methods

### Media and chemicals used

Oxoid and Difco were used to get the media preparation in the biological activities. Chemicals used in the bimetallic NPs synthesis such as zinc nitrate hexahydrate (Zn(NO_3_)_2_·6H_2_O), boric acid (H_3_BO_3_), isopropanol ((CH_3_)_2_CHOH), and gum arabic were purchased from Sigma-Aldrich (UK) which were considered as standard ingredients.

### Gamma radiation

Environmentally friendly techniques including gamma irradiation^28–30^ were used at the NCRRT in Cairo, Egypt. The materials were gamma-irradiated in solution form using the Co-Gamma Chamber 4000-A-India as the radiation source. After dissolving the first precursors, the radiation time was calculated to be 1.036 kGy per hour (dose rate).

### Synthesis of bimetallic B_2_O_3_–ZnO NPs

The biogenesis of B_2_O_3_–ZnO NPs used the precise quantity of salts. More specifically, 10 ml of (2.0 mM) Zn(NO_3_)_2_·6H_2_O and 10 ml of (2.0 mM) H_3_BO_3_ were mixed for about 30 min at room temperature. Additionally 80 ml of the prepared gum arabic solution (directed as stabilizing agent) was then added to them. After preparing the combined solution, we measured the pH of the mixture and noted that it was 7.2. In order to achieve the most effective synthesis of B_2_O_3_–ZnO NPs, the conditions of the reaction were determined to be an incubator shaking at 500 rpm for approximately 24 h while maintaining an incubation temperature of 30 °C.^31^ After the incubation period was complete, we exposed the produced solution to a dosage of 20 kGy gamma rays (directed as a reducing agent), which was chosen in accordance with our published and related publications.^19,32^

We observed the color change after the gamma irradiation process and recorded it as off-white (as shown in Fig. S1†), confirming the formation of B_2_O_3_–ZnO NPs. The produced B_2_O_3_–ZnO NPs were then centrifuged at 5000 rpm for about 20 minutes to remove any leftover gum arabic biomolecules after being washed five times with distilled water.

### Characterization of bimetallic B_2_O_3_–ZnO NPs

The optical characteristics of the tested bimetallic B_2_O_3_–ZnO NPs were investigated using a UV-Vis spectrophotometer (JASCO V-560) with a specific wavelength range of 190 to 900 nm. Using dynamic light scattering (DLS-PSS-NICOMP 380, USA), the particle size distribution of the produced bimetallic B_2_O_3_–ZnO NPs was determined. The HR-TEM, JEM2100, Jeol, Japan, was employed to ascertain the precise generated shape and the mean and correct particle size to prove their production at the nanomaterials scale. The XRD-6000 (Shimadzu Scientific Instruments, Japan) used XRD analysis to confirm the accurate development of the crystalline materials. XRD analysis was used to establish the crystal size of the resulting bimetallic B_2_O_3_–ZnO NPs. The last stage was utilizing a SEM, ZEISS, EVO-MA10, Germany, to assess the surface quality and exact out surface form of the synthesized bimetallic B_2_O_3_–ZnO NPs. Finally, at the pH of synthesis, the surface charges of the synthesized bimetallic B_2_O_3_–ZnO NPs were indirectly evaluated using a Malvern device zeta potential analyzer, UK.

### Cytotoxicity and anticancer activity

Bimetallic B_2_O_3_–ZnO NPs cytotoxicity was assessed using a slightly modified version of the MTT technique.^33,34^ The American Type Culture Collection (ATCC) provided normal Vero, Wi38 normal cell lines and the malignant Caco 2 (Caucasian colon adenocarcinoma) cell line. Tissue culture plate (96 well) was inoculated with 1 × 10^5^ cells per ml (100 μl per well) and incubated at 37 °C for 24 h to develop a complete monolayer sheet. Growth medium was decanted from 96 well micro titer plates after confluent sheet of cells were formed, cell monolayer was washed twice with wash media. Two-fold dilutions of tested sample was made in RPMI medium with 2% serum (maintenance medium). Then, 0.1 ml of each dilution was tested in different wells leaving 3 wells as control, receiving only maintenance medium, then plate was incubated at 37 °C and examined. Cells were checked for any physical signs of toxicity, *e.g.* partial or complete loss of the monolayer, rounding, shrinkage, or cell granulation. MTT solution was prepared (5 mg ml^−1^ in PBS) (Bio Basic Canada Inc.), where 20 μl MTT solution were added to each well then shook at 150 rpm for 5 min, then followed by incubation at 37 °C, 5% CO_2_ for 4 h to allow the MTT to be metabolized. The optical density was measured at 560 nm. The following formula (1) was used to calculate the viability of cells, while eqn (2) was applied for cell inhibition determination:1
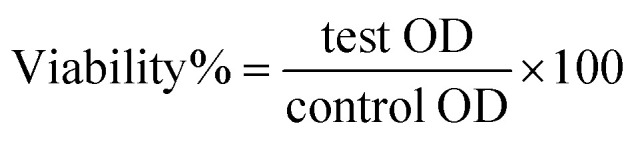

2 Inhibition% = 100 − viability%

### Antimicrobial activity

Antimicrobial activity of bimetallic B_2_O_3_–ZnO NPs was assessed against Gram-negative bacteria (*Escherichia coli* ATCC 25922 & *Pseudomonas aeruginosa* ATCC 27853), Gram-positive bacteria (*Staphylococcus aureus* ATCC 25923 & *Bacillus subtilis* ATCC 6051), and unicellular fungi (*Candida albicans* ATCC 90028). With a few minor modifications, the agar well diffusion procedure was carried out in accordance with document M51-A2 of the Clinical Laboratory Standard Institute.^35^ One hundred microliter of each bimetallic B_2_O_3_–ZnO NPs, zinc salt, boron salt, standard antibiotic (norfloxacin) and antifungal drug (fluconazole) at concentration of 1000 μg ml^−1^ was put in agar well (7 mm) seeded plates with bacterial and fungal strains individually and incubated at 37 °C for 24–48 h, then inhibition zones were measured.^36–38^ To determine minimum inhibitory concentration, microdilution method was used.^39–41^

### Leakage effect of bimetallic B_2_O_3_–ZnO NPs on *S. aureus*

The effect of bimetallic B_2_O_3_–ZnO NPs on membrane leakage of treated *S. aureus* with 2 × MIC of bimetallic B_2_O_3_–ZnO NPs was determined at intervals incubation periods (0, 6, 12, 18, 24 and 30 h). To detect the leakage of total proteins through *S. aureus* membrane, 100 μl from fresh *S. aureus* culture was mixed with 2 × MIC bimetallic B_2_O_3_–ZnO NPs and incubated at 37 °C with shaking at 150 rpm. Control experiments were included without B_2_O_3_–ZnO NPs. *S. aureus* culture (1 ml) was centrifuged at 10 000 rpm, the supernatant was frozen at −30 °C immediately, and then the total proteins was measured.^42^ For determination of lipid peroxidation in cell membrane, *S. aureus* was treated with 2 × MIC B_2_O_3_–ZnO NPs at different incubation periods (0, 6, 12, 18, 24 and 30 h). About 6% (w/v) trichloroacetic acid (TCA) was added to treated cell suspensions to precipitate the cell proteins followed by incubation at room temperature, for 30 min. Centrifugation at 10 000 rpm for 30 min was performed to samples and 1% aqueous thiobarbituric acid (TBA) (Sigma-Aldrich, >98%) solution was added in a ratio of 1 : 1. The mixture was boiled for 30 min, cooled to room temperature, for overnight and measured using a UV-visible spectrophotometer at 532 nm.^43,44^

### Antioxidant activity

Bimetallic B_2_O_3_–ZnO NPs were tested for antioxidant activity using the DPPH (2,2-diphenyl-1-picrylhydrazyl) technique.^45^ Different concentrations of bimetallic B_2_O_3_–ZnO NPs (1000, 500, 250, 125, 62.5, 31.25, 15.62, 7.81 μg ml^−1^) were used to determine the ability to scavenge DPPH radicals. The method used by ref. 9 was carried out to evaluate antioxidant activity of ascorbic acid (AA) and different concentrations of bimetallic B_2_O_3_–ZnO NPs were determined as DPPH scavenging activity (%) calculated by the following eqn (3):3
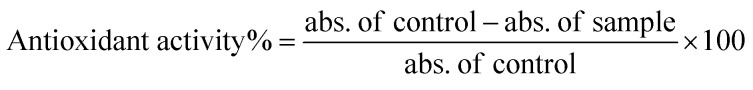


## Results and discussion

### Proposed reaction mechanism for the synthesis of bimetallic B_2_O_3_–ZnO NPs

In the current work, reduction was most successful at 20.0 kGy, indicating that gamma radiation has a crucial role in the synthesis of B_2_O_3_–ZnO NPs as shown in Table 1. Kinetic analyses demonstrated that metal ions reduction to NPs always commences with gamma irradiation initiation in aqueous medium.^32^

**Table 1:** Proposed reaction mechanism regarding bimetallic B_2_O_3_–ZnO NPs synthesis

Reaction inputs	Condition	Products	Equation
H_2_O	Radiolysis (γ-ray)	e_aq_^−^, OH˙, H˙, H_2_, and H_2_O_2_	(4)
Zn(NO_3_)_2_ + H_2_O	Hydrolysis	2Zn^+^ + 2NO_3_^−^	(5)
B(OH)_3_ + H_2_O		BOH_4_^−^ + H_3_O^+^	(6)
Zn^+^ + e_aq_^−^	Reduction	Zn NPs (not stable)	(7)
BOH_4_^−^ + e_aq_^−^		B NPs (not stable) + 4OH^−^	(8)
BOH_4_^−^ + 4Zn^+^	Complexation	Zn–BOH_4_	(9)
GA + OH˙ (and/or H˙)	H-abstraction	GA˙ (and GA˙˙) + H_2_O (and/or H_2_)	(10)
GA˙ + Zn^+^ + H_2_O	Reduction & capping	GA-capped Zn NPs + H_3_O^+^	(11)
GA˙ + BOH_4_^−^ + H_2_O		GA-capped B NPs + 4H_2_O	(12)
GA + 2Zn–BOH_4_ + H_2_O		GA-capped ZnO–B_2_O_3_ NPs + H_2_O	(13)
GA˙˙ + Zn^+^ + 2BOH_4_^−^ + 2H_2_O		GA-capped ZnO–B_2_O_3_ NPs + H_3_O^+^	(14)

After being exposed to gamma radiation, water created a variety of radical species, including solvated electrons (e_aq_^−^), OH˙, H˙, H_2_O_2_, and H_2_, according to eqn (4). The creation of highly reducing free radicals, or e_aq_^−^, that carry out their task without generating any unnecessary byproducts was a benefit of gamma irradiation for the synthesis of bimetallic NPs.^46,47^ In order to create nanoparticles, Zn(NO_3_)_2_ and H_3_BO_3_ were first dissolved, resulting in the hydrated cations Zn^+^ and H^+^ and the anions BOH_4_^−^ and NO_3_^−^ (eqn (5) and (6) in Table 1).^48,49^ After then, according to eqn (7) and (8) there is a chance that both Zn^+^ and BOH_4_^−^ will be obviously reduced by e_aq_^−^, creating non-capped ZnO NPs and B_2_O_3_ NPs that are prone to disintegrate.^50^ In simultaneously eqn (9) in Table 1 shows that the likely interaction of Zn^+^ with BOH_4_^−^ produced the Zn–BOH_4_ complex.^51^

Furthermore, GA˙ and GA˙˙ radicals were produced when OH˙ and H˙ radicals interacted with the hydrogen atoms in GA (eqn (10)).^52^ Eqn (11) and (12) predict that GA˙ and GA˙˙ may each separately react with Zn^+^ and BOH_4_^−^ to create stable-capped ZnO NPs and B_2_O_3_ NPs.

In addition, one of two processes: (i) GA interaction with Zn–BOH_4_ complex, eqn (13) and (ii) simultaneous reduction of Zn^+^ and BOH_4_^−^ by the single moiety GA˙˙ which produced stable-capped bimetallic B_2_O_3_–ZnO NPs using the bi-radical positions eqn (14).

Surface plasmon resonance (SPR), which is caused by electron excitation in the conduction zone outside B_2_O_3_–ZnO, is a phenomenon.^53^ The dimension and shape of the particles affect the distinctive oscillation features. It is important to note that light electromagnetism crosses the free electrons, more especially the conduction line-located electrons of zinc and/or boron ions, to form fused combined flow after activation of inorganic NPs by a light source.^54^

The whole reaction demonstrated the importance of GA as a stabilizing polymer for the formation of B_2_O_3_–ZnO NPs as well as the involvement of electrons in decreasing Zn^+^ and BOH_4_^−^ ions. The average particle size and particle size distribution of the manufactured ZnO NPs, B_2_O_3_ NPs, and B_2_O_3_–ZnO NPs were raised by increasing the dosage of gamma rays up to 20.0 kGy.

That was ascribed to the aggregation and precipitation of the synthesized ZnO NPs, B_2_O_3_ NPs, and B_2_O_3_–ZnO NPs under the influence of extra electrons and free radicals produced during water radiolysis by gamma rays through the process shown in eqn (4).^55–58^

### Characterization of bimetallic B_2_O_3_–ZnO NPs

The capacity of the gamma rays to synthesize B_2_O_3_–ZnO NPs in the presence of a capping gum arabic was evaluated. The prepared solution first seemed to be faint white color, but as bimetallic B_2_O_3_–ZnO NPs were synthesized, the color changed to a deep off-white (Fig. S1†). A valid spectroscopic indication of their appearance was supplied by the produced off-white hue, which was attributable to the activation of the surface plasmon resonance of bimetallic B_2_O_3_–ZnO NPs.^59^

**Fig. 1:**
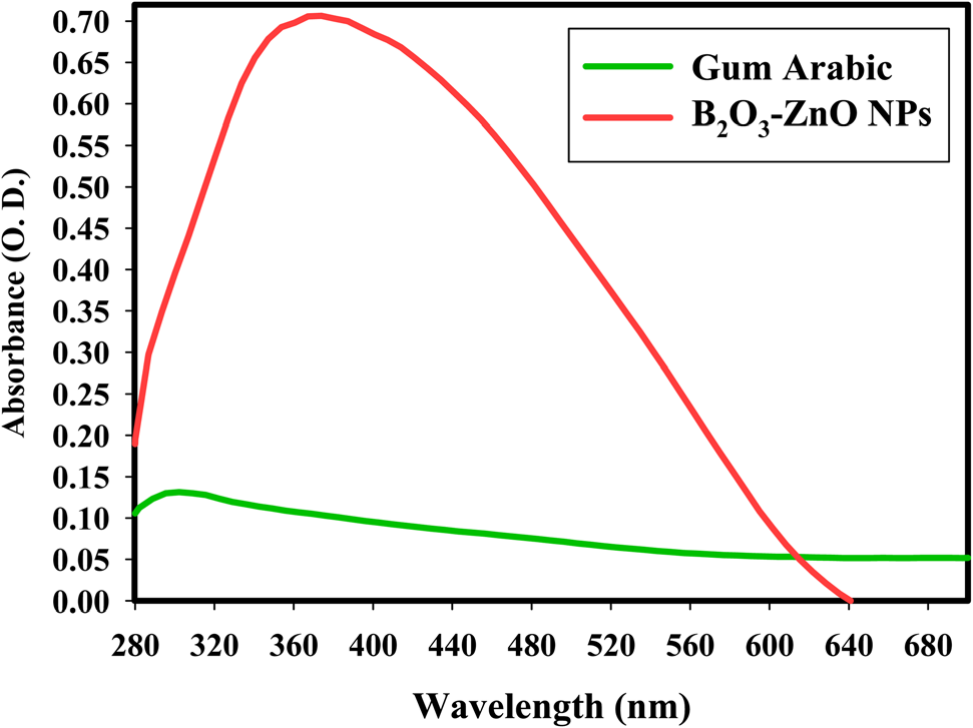
UV-Vis spectrum of bimetallic B_2_O_3_–ZnO NPs (diluted 5 times).

Due to the O.D. (0.709; diluted five times), the experimental peak was evident in the spectra (Fig. 1). The UV-Vis investigations showed that the generated B_2_O_3_–ZnO NPs were tiny and visible at 370.0 nm. The UV-Vis spectra of the produced gum arabic demonstrates the presence of a distinct peak and is consistent with literature findings.^60,61^

The off-white color's intensity matched the produced capacity to synthesize B_2_O_3_–ZnO NPs.^62,63^ The strength, size, morphological surfaces, structure, and dielectric properties of any generated nanoparticles have a significant impact on surface plasmon resonance (SPR).^64,65^

After comparison with publications in the literature on intermediate particle size and form, it was found that the synthesized bimetallic B_2_O_3_–ZnO NPs were poly-dispersed, varied in size, and mainly had spheroidal particles as their predominant shape. Wide-ranging forms may have been developed in that work.^66^ Although all of the newly produced NPs were sphere- or orbicular-shaped, different morphologies may have been seen as a result of the extraction-based synthetic process, which is why the anisotropic form had been detected. A stable form is poly-displayed NPs, since only the most practical reducing (gamma rays) and capping agent (gum arabic) were utilized in our work.

**Fig. 2:**
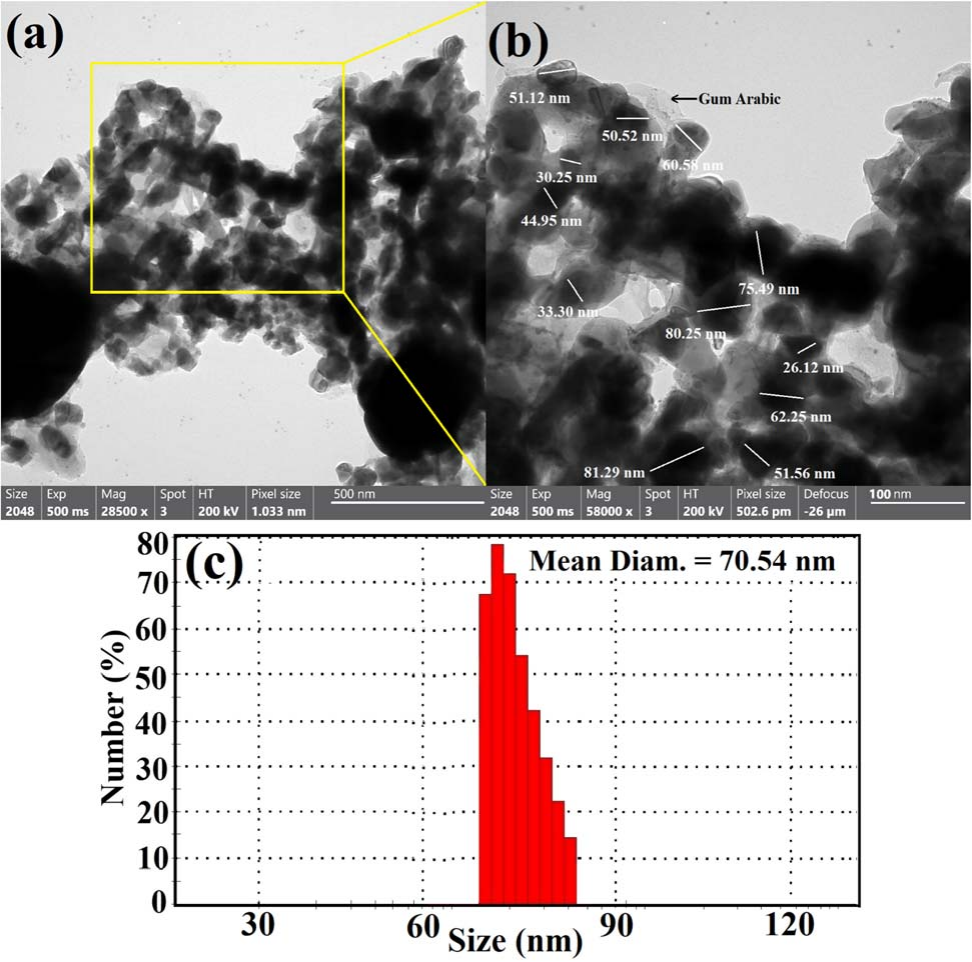
HRTEM imaging (a and b) and DLS analysis (c) of the synthesized bimetallic B_2_O_3_–ZnO NPs.

DLS was used to study the hydrodynamic radius, particle size distribution, and polydispersity index (PDI) of synthesized bimetallic B_2_O_3_–ZnO NPs. To ascertain the typical size of these nanoparticles, the acquired data were compared to the HRTEM research.^67^ The HR-TEM image in Fig. 2a for the synthetic bimetallic B_2_O_3_–ZnO NPs revealed that the particles were semi-spherical and attached with the stabilizing gum arabic as shown in Fig. 2a, and their sizes ranged from 26.12 nm to 81.29 nm, with an average diameter of 53.97 ± 2.0 nm (Fig. 2b). The provided poly-dispersed NPs were intended to decrease, stabilize, and act as capping agents for the generated gum arabic filtrate that was rich in active functional groups, among other things.^68^

The line spacing was exactly the same, resulting in one grade system, as shown by the HRTEM image result (Fig. 2b). It demonstrated that boron was uniformly distributed throughout the zinc matrix, producing a unique alloy. Similarly, the prepared radical-multi-position of gum arabic may generate concurrent decrease of Zn and B.^69^ According to the DLS method, the typical particle size distribution for bimetallic B_2_O_3_–ZnO NPs, which were synthesized by gamma rays and gum arabic, was calculated to be 70.54 nm as exhibited in Fig. 2c.

According to International Standards Organizations (ISOs), samples are deemed to be monodisperse when the polydispersity index (PDI) findings are less than 0.05. In contrast, PDI outcomes of greater than 0.7 are intended to produce particles with a polydispersity distribution.^70^ Based on our findings, the PDI values for bimetallic B_2_O_3_–ZnO NPs were 0.89. According to the current values, the synthesized bimetallic B_2_O_3_–ZnO NPs were a reasonable range of polymers. The findings showed that the estimated sizes of the particles identified by HRTEM imaging were smaller than the mean and prevalent sizes indicated by DLS analysis. The hydrodynamic radius within the bimetallic B_2_O_3_–ZnO NPs and the water layers surrounding them are the reasons for the substantial diameters of the synthesized bimetallic B_2_O_3_–ZnO NPs.^71^

**Fig. 3:**
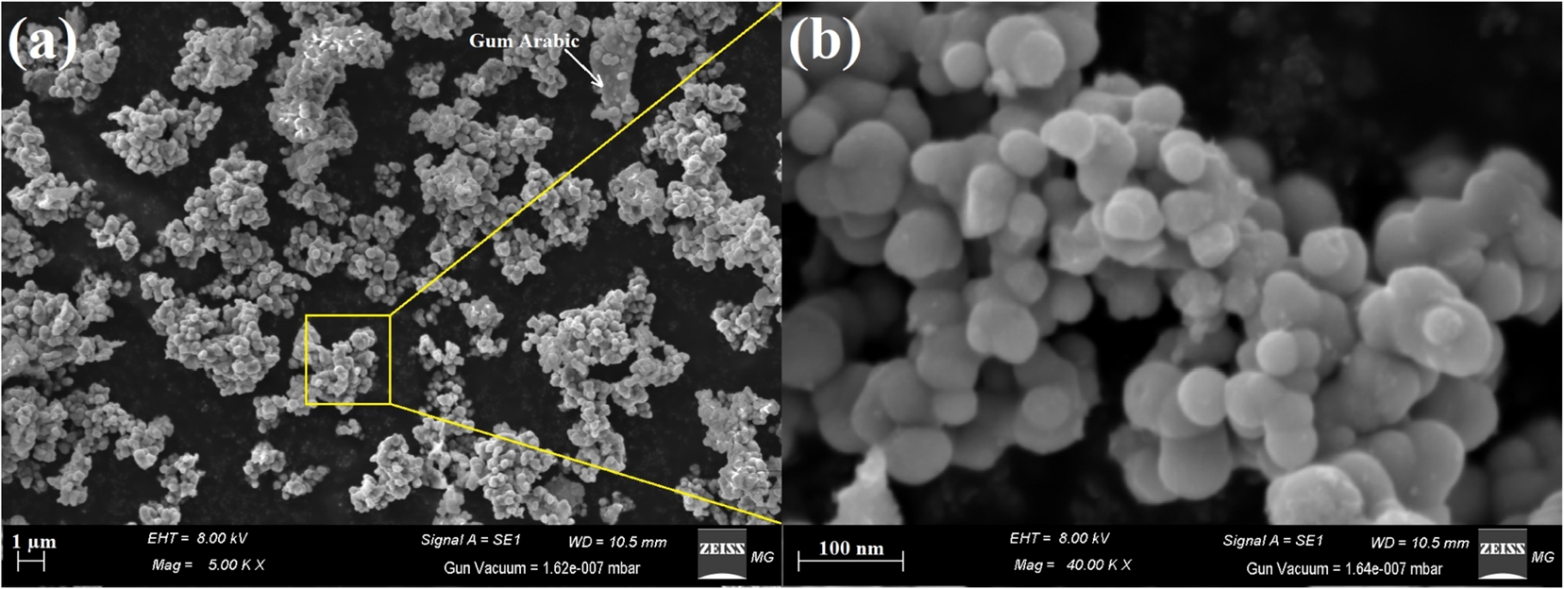
SEM images of the synthesized bimetallic B_2_O_3_–ZnO NPs (a), and the magnified SEM image (b).

The surface properties and surface shape of the synthesized B_2_O_3_–ZnO NPs were investigated using the SEM method. SEM results for mixed B_2_O_3_–ZnO NPs and newly made gum arabic show consistent B_2_O_3_–ZnO NP surfaces with a clear surface appearance (Fig. 3a). The produced gum arabic had the identical brilliant spherical particles. When gum arabic was formed, which appears as an illuminated NPs merged and capped with it, it was found that B_2_O_3_–ZnO NPs were effectively separated as spheroidal particles fused with one another across it (Fig. 3b).

We compared the morphological form of the synthesized B_2_O_3_–ZnO to others reported in the literature. Here, NPs were evenly dispersed with restricted size and the perfect spherical formation.

At various pH and temperature conditions, Muhammad *et al.*^72^ used the citrate reduction technique to synthesize bimetallic silver and gold core–shell NPs. The pH and temperature play a crucial role in the synthetic process since the approved morphological shape and border size advised that they have maintained size ranges between 50 and 65 nm and appear as spheroidal particles.

**Fig. 4:**
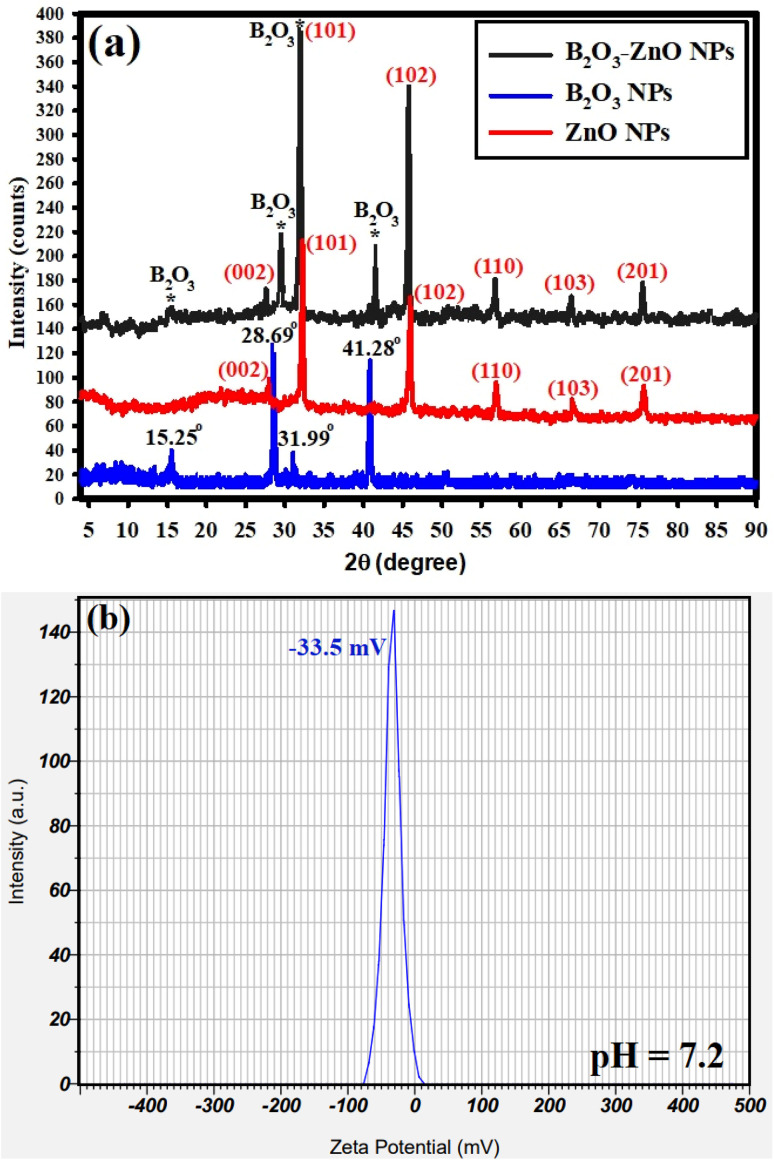
XRD analysis (a), and zeta potential determination (b) of the bimetallic B_2_O_3_–ZnO NPs.

The XRD analyses for the synthesized bimetallic B_2_O_3_–ZnO NPs are shown in Fig. 4a. The produced NPs depict the amorphous and crystal arrangements of the precursor (gum arabic) and synthesized bimetallic B_2_O_3_–ZnO NPs, respectively. In order to directly detect the distinct lines of the synthesized B_2_O_3_–ZnO NPs, we analyses the XRD data of our previously synthesized ZnO NPs, and B_2_O_3_ NPs as separated spectrum (Fig. 4a). It should be emphasized that 2*θ* refers to the gum arabic in the 2*θ* range of 5° to 25°.^69,73^Fig. 4a displays the XRD results of the produced bimetallic B_2_O_3_–ZnO NPs and emphasizes the diffraction peaks of the ZnO NPs. These peaks include those at 2*θ* = 27.50°, 31.15°, 45.15°, 56.89°, 67.98°, and 75.25°. These peaks, which are complemented with the standard card JCPDS number 361451, correspond to (002), (101), (102), (110), (103), and (201) Bragg's reflections, respectively.^74^ They also contain the B_2_O_3_ NPs diffraction peaks at 2*θ* = 15.25°, 28.69°, 31.99°, and 41.28°, which are complemented by the usual card JCPDS number 300019.^75^

The available XRD data (Fig. 4a) shows that the synthesized B_2_O_3_–ZnO NPs were crystallized and had a face-centered cubic (fcc) crystalline structure. The generated bimetallic NPs were highly crystalline and coupled with amorphous gum arabic, improving their diffusion in the solution for improved biomedical application, according to the XRD data.^76^

Finally, the equation of Williamson–Hall (W–H) was used to define the intermediate crystallite size of bimetallic B_2_O_3_–ZnO NPs,^77,78^ and provided to eqn (15) were found to be 35.35 nm.15
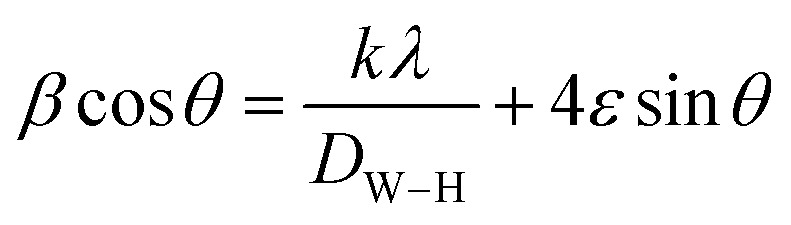


As shown in Fig. 4b, the zeta potential of the synthesized bimetallic B_2_O_3_–ZnO NPs was investigated at the pH of the synthesis (7.2). The surface zeta potential of the synthesized bimetallic B_2_O_3_–ZnO NPs maintains a negative statement at the pH of the synthesis that was examined, according to the current study. Additionally, because of the negative charge of gum arabic, the preparation's zeta potential at neutral medium (pH 7.2) was −33.5 mV, as illustrated in Fig. 4b.

### Anticancer activity

**Fig. 5:**
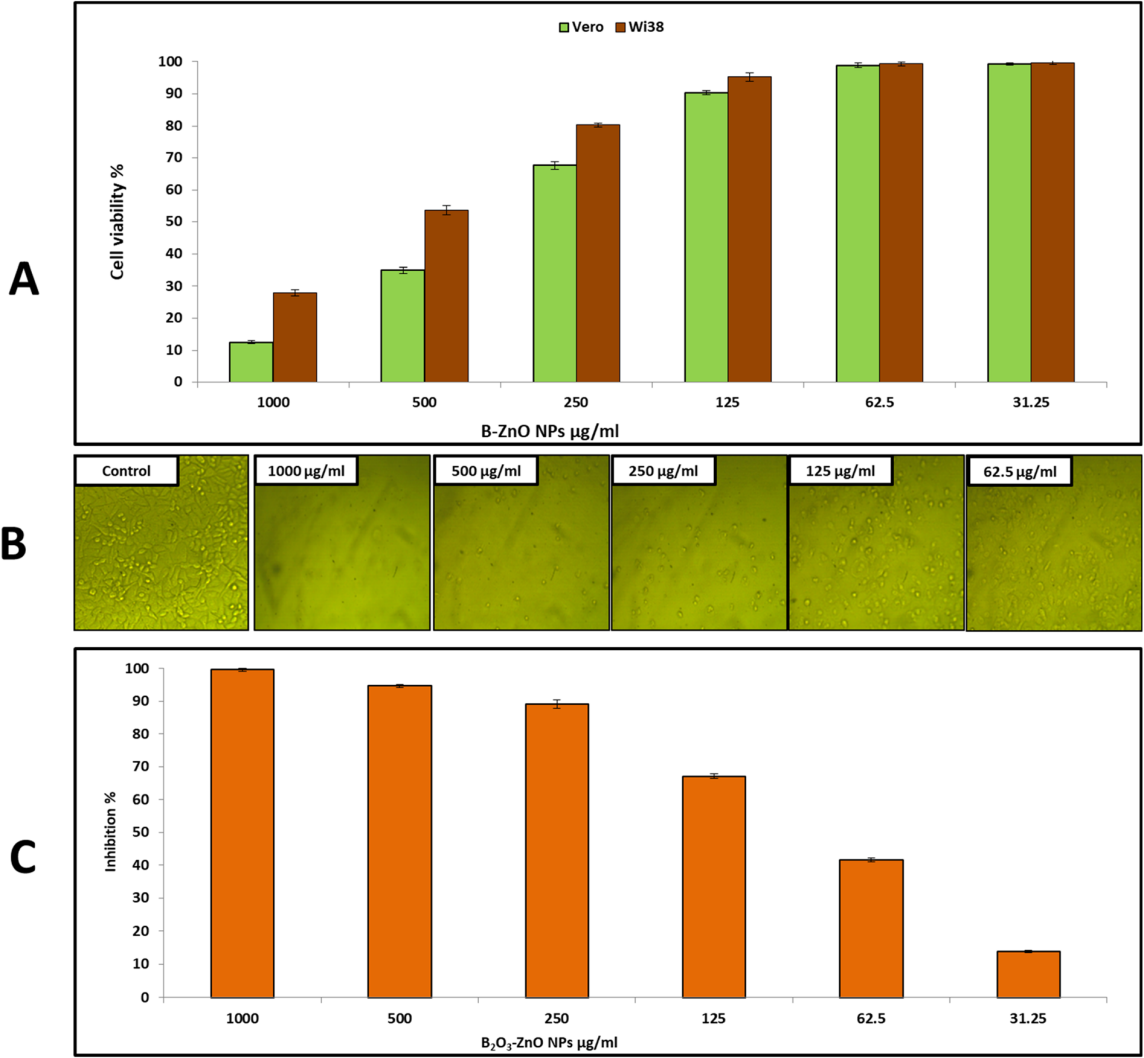
Effect of bimetallic B_2_O_3_–ZnO NPs on cell viability of Vero, and Wi38 normal cell line (A) and cell inhibition of Caco 2 cell line (B and C).

Cytotoxicity against the Vero and Wi38 normal cell line was assessed to determine the safety of bimetallic B_2_O_3_–ZnO NPs, as shown in Fig. 5A. The results showed that cell viability of Vero cells was 99.26, 98.8, 90.3, and 67.6% at doses of 31.25, 62.5, 125, and 250 μg ml^−1^, respectively. Also, cell viability of Wi38 was 99.6, 99.3, 95.2, 80.3 and 53.6% at doses of 31.25, 62.5, 125, 250 and 500 μg ml^−1^, respectively. Furthermore, IC_50_ of bimetallic B_2_O_3_–ZnO NPs toward Vero and Wi38 normal cell line was 384.5 and 569.2 μg ml^−1^, this confirms that bimetallic B_2_O_3_–ZnO NPs are safe in use due to if the compound is classified as non-cytotoxic when IC_50_ is ≥90 μg ml^−1^.^79^

On the other hand, cancerous Caco 2 cell line was used to assess anticancer activity of bimetallic B_2_O_3_–ZnO NPs at different concentrations as shown in Fig. 5B and C. Our results illustrated that, bimetallic B_2_O_3_–ZnO NPs displayed promising anticancer activity toward cancerous Caco 2 where IC_50_ was 80.1 μg ml^−1^. Furthermore, inhibition percentages of Caco 2 at concentrations 1000, 500, 250, 125 and 62.5 μg ml^−1^ were 99.6, 94.7, 89.2, 67.0 and 41.6%, respectively. Previous studies reported that bimetallic nanoparticles have promising cytotoxic activities against cancerous cell lines.^80–85^ Moreover, both cancerous MCF-7 and Caco 2 cell lines were used to evaluate bimetallic Ag–ZnO NPs, where bimetallic Ag–ZnO had promising anticancer activity, and IC_50_ were 104.9 and 52.4 μg ml^−1^, respectively.^81^

Additionally, bimetallic ZnO–Ag NPs were fabricated using laser ablation and results confirmed that bimetallic ZnO–Ag NPs have anticancer activity against the malignant cell lines HCT 116 and HeLa.^83^ Moreover, bimetallic Ag–Au NPs shown anticancer activity against malignant HT-29 and MCF-7 cell lines, according to ref. 85. Also, Alafaleq *et al.*,^86^ reported that the biosynthesized bimetallic Cu–Mn NPs had anticancer activity toward HT-29 cell line with an IC_50_ dose of 115.2 μg ml^−1^.

Generally, anticancer activity of nanoparticles attributed to different mechanisms such as ROS induced-apoptosis which is considered one of the main anticancer mechanisms which damage cell membrane, dysfunction of mitochondria, oxidation of enzymes and proteins, DNA fragmentation,^87^ up- and down-regulation of apoptotic regulatory proteins inducing programmed cell death.^88^

### Antimicrobial activity

**Fig. 6:**
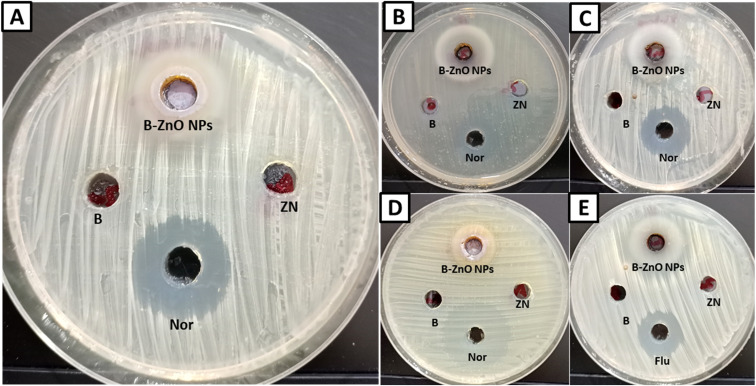
Inhibition zones of bimetallic B_2_O_3_–ZnO NPs against *E. coli* (A), *P. aeruginosa* (B), *B. subtilis* (C), *S. aureus* (D) and *C. albicans* (E) (B for boric acid, Zn for zinc nitrate, Nor for norfloxacin, Flu for fluconazole, and B–ZnO NPs for bimetallic B_2_O_3_–ZnO nanoparticles).

Bimetallic nanoparticles feature distinctive geometrical architecture and mixing patterns, which improve their functionality.^89^ They outperform monometallic nanoparticles in terms of stability, selectivity, and catalytic activity.^90^ In the current study, antimicrobial activity of the synthesized bimetallic B_2_O_3_–ZnO NPs was assessed against *E. coli*, *P. aeruginosa*, *B. subtilis*, *S. aureus* and *C. albicans* (Fig. 6 and Table 2). Results revealed that the synthesized bimetallic B_2_O_3_–ZnO NPs exhibited antibacterial activity toward both Gram-positive and Gram negative bacteria as well as antifungal activity against unicellular fungi. Moreover, B_2_O_3_–ZnO NPs displays promising antibacterial against *E. coli*, *P. aeruginosa*, *B. subtilis* and *S. aureus* where inhibition zones at concentration 1000 μg ml^−1^ were 21.80 ± 1.71, 27.97 ± 1.55, 20.67 ± 1.53 and 28.03 ± 0.95 mm, respectively.

**Table 2:** Antimicrobial activity of bimetallic B_2_O_3_–ZnO NPs against *E. coli*, *P. aeruginosa*, *B. subtilis*, *S. aureus* and *C. albicans*

Microorganism used	Zinc nitrate	Boric acid	B_2_O_3_–ZnO NPs		NOR/FLUC^a^	
	IZ	IZ	IZ	MIC	IZ	MIC
*E. coli*	ND^a^	ND	21.80 ± 1.71	125	24.00 ± 1.00	125
*P. aeruginosa*	ND	ND	27.97 ± 1.55	62.5	19.83 ± 0.76	250
*B. subtilis*	ND	ND	20.67 ± 1.53	125	31.93 ± 1.10	31.25
*S. aureus*	ND	ND	28.03 ± 0.95	62.5	24.93 ± 0.90	125
*C. albicans*	ND	ND	27.33 ± 0.58	62.5	21.70 ± 0.52	250

a ND means not detected, NOR/FLUC for norfloxacin, and fluconazole as antimicrobial standard.

Also, MIC was determined for each test organism, where MICs of B_2_O_3_–ZnO NPs toward *E. coli*, *P. aeruginosa*, *B. subtilis* and *S. aureus* were 125, 62.5, 125 and 62.5 μg ml^−1^, respectively.

Likewise, B_2_O_3_–ZnO NPs had potential antifungal activity against *C. albicans* as unicellular fungi where inhibition zone was 28.03 ± 0.95 mm, also MIC was 62.5 μg ml^−1^. According to cytotoxicity results, these concentration 62.5–125 μg ml^−1^ are safe use due to IC_50_ of Vero and Wi38 normal cell line was 384.5 and 569.2 μg ml^−1^, respectively.

Bimetallic nanoparticles can work in conjunction with antibiotics to battle microorganisms. These nanoparticles can obstruct bacterial membrane function or produce ROS (reactive oxygen species), which can damage DNA and impair the functioning of bacterial proteins.^91^ The interaction between positively charged NPs and negatively charged bacterial cell membranes induced oxidative stress and amplifies bacterial protein degradation can explain the highest antibacterial activity of bimetallic nanoparticles.

Additionally, metallic ions disrupt hemostasis by attaching to the SH groups of the peptidoglycan layer, which weakens the cell wall. Additionally, metallic nanoparticles may collect on bacterial cell membranes and generate ROS, which consequentially induces bacterial cells death. Bimetallic Ag–Au NPs synthesized from *Gracilaria* sp. exhibited antibacterial activity against *S. aureus* and *Klebsiella* sp.^92^ In addition, *P. aeruginosa*, *S. aureus*, *B. subtilis*, and *E. coli* are susceptible to Au–Ag bimetallic nanoparticles derived from the flower and leaf extracts of *Ocimum basilicum* (basil).^93^ In addition, a recent report stated that the produced eco-friendly and cost-effective bimetallic Ag–ZnO NPs may be used as anticancer and antimicrobial agents in the biomedical domains.^81^ In a new paper bimetallic Cu–Zn NPs shown antibacterial activity of stem extracts of *Cissus quadrangularis* and proneness against *E. coli*, *P. aeruginosa*, *B. subtilis*, *S. aureus* and *S. mutans*.^94^ Furthermore, bimetallic ZnO–Ag nano encapsulated in PVP/PCL nano-fibers was fabricated and found this nano-compound has promising antibacterial activity against *S. aureus* and *E. coli* better than monometallic loaded nanofibers.^95^

Also, a recent work have biosynthesized bimetallic Ag–Cu and Cu–Zn NPs and exhibited antibacterial activity toward *Alcaligenes faecalis*, *S. aureus*, *Citrobacter freundii*, *Klebsiella pneumoniae* and *Clostridium perfringens*.^96^ Additionally, the biosynthesized Ag–ZnO NPs had antibacterial activity where MIC was 0.125 μg ml^−1^ against *S. aureus* and *B. subtilis*.

### Cell membrane leakage of *S. aureus*

**Fig. 7:**
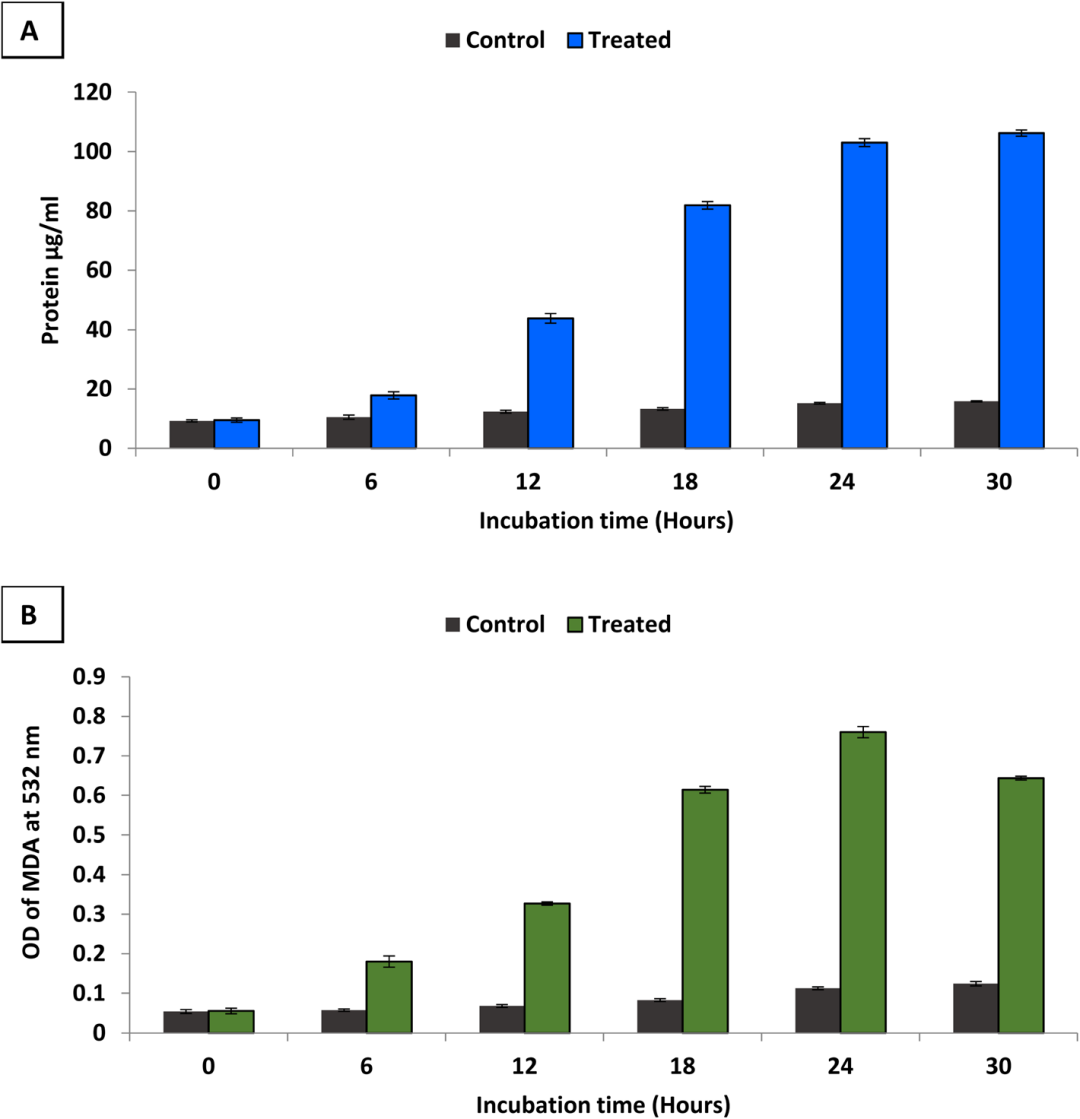
Leakage of protein (A) and lipid (B) from treated *S. aureus* with bimetallic B_2_O_3_–ZnO NPs (2 × MIC).

To study antimicrobial mechanism of the synthesized bimetallic B_2_O_3_–ZnO NPs, *S. aureus* was selected for this purpose due to it was the most sensitive bacteria among others. The ability of bimetallic B_2_O_3_–ZnO NPs to damage the integrity of the cell membrane of *S. aureus* was evaluated by measuring the total proteins released in treated cell suspensions as well as determining lipid peroxidation inside the bacterial cell. In the current study, some contents of *S. aureus* cell membrane as proteins and lipid peroxidation were determined in the case of treating with bimetallic B_2_O_3_–ZnO NPs (2 × MIC) as illustrated in Fig. 7.

Results revealed that, the amount of protein released from the *S. aureus* increased along with increasing the incubation time of B_2_O_3_–ZnO NPs (Fig. 7A). At 6 h, the treated cells leaked approximately 17.85 μg ml^−1^ of protein, and leakage percentage increased gradually with increasing incubation time where protein reached to 81.9 μg ml^−1^ at 18 h. Moreover, the protein increased approximately ten times at 24 h where was 103.05 μg ml^−1^. Furthermore, results showed non-significant increase in protein where was 106.25 μg ml^−1^ at 30 h. Thus, incubation time between 24 and 30 h was the best for protein leakage from treated *S. aureus* with bimetallic B_2_O_3_–ZnO NPs.

Furthermore, lipid peroxidation was determined by measuring malondialdehyde (MDA) concentration which is one of the most important degradation products of lipid peroxidation.^97^ TBA was used to detect MDA concentration, where TBA reacts with MDA to give MDA–TBA adduct (pink color). Fig. 7B shows determination of MDA–TBA adducts for control and treated *S. aureus* at different incubation times (6, 12, 18, 24 and 30 h). Results showed that, appearance high amounts of MDA in treated cells at different times comparable to control which indicate bimetallic B_2_O_3_–ZnO NPs affect directly on cell membrane fatty acids of *S. aureus*. Also, results showed that the highest lipid peroxidation percentage was at incubation time 24 h in compared to control as well as other different incubation times. Zinc oxide nanoparticles generated ROS and induced lipid peroxidation in the liposomal membrane of Gram-positive bacteria.^98^ Likewise in other study, ZnO NPs exhibited antibacterial activity through generating ROS which induced lipid peroxidation.^99^

### Antioxidant activity

**Fig. 8:**
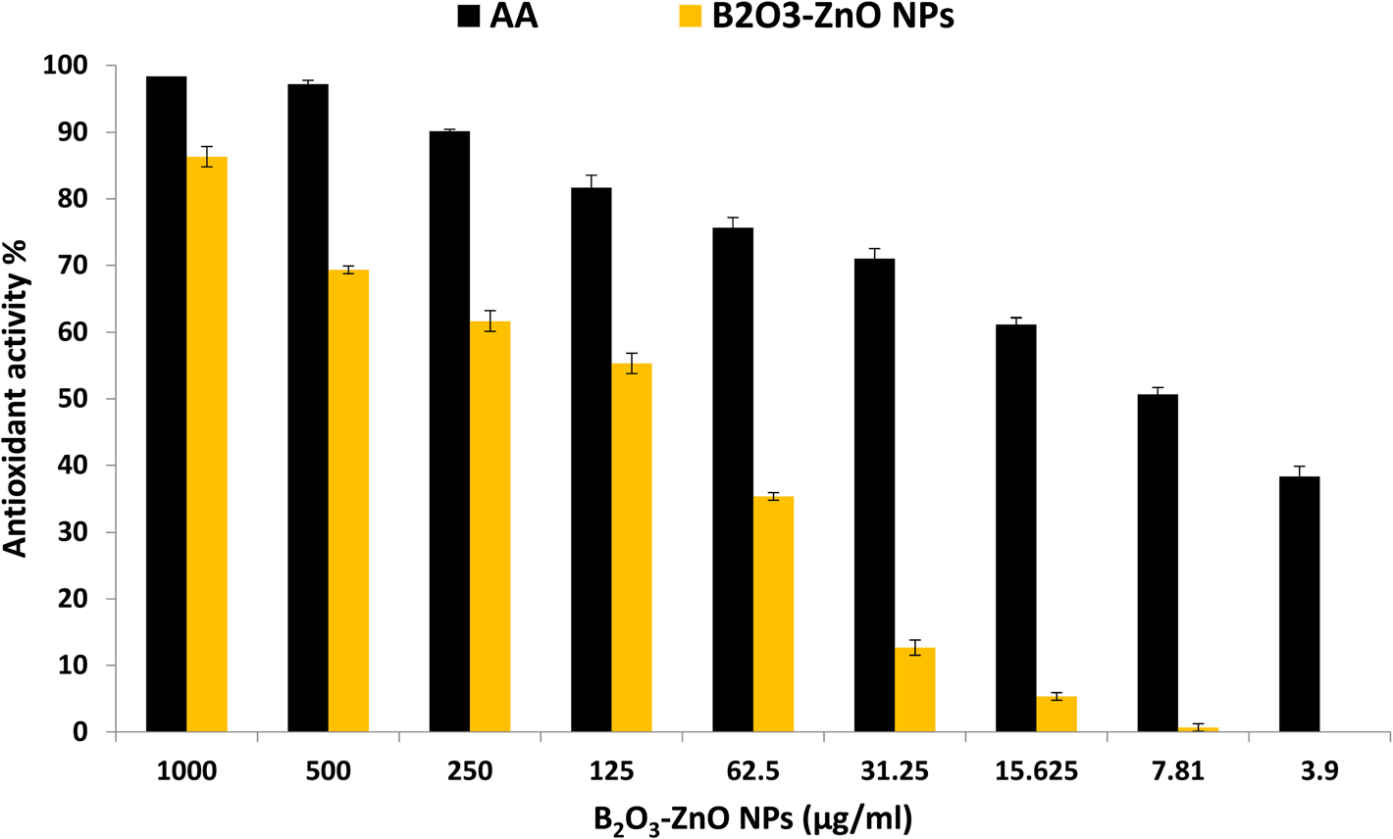
Antioxidant activity of bimetallic B_2_O_3_–ZnO NPs at different concentration using DPPH method.

According to Phaniendra *et al.*,^100^ the interaction of biomolecules with molecular oxygen results in the production of free radicals in biological systems. In addition, antioxidants have been suggested as therapeutic agents because of their anti-atherosclerotic, anti-inflammatory, anti-cancer, anti-mutagenic, and antibacterial characteristics.^101,102^ In the current study, antioxidant activity of bimetallic B_2_O_3_–ZnO NPs at different concentrations was evaluated as shown in Fig. 8. Results revealed that bimetallic B_2_O_3_–ZnO NPs have antioxidant activity where IC_50_ was 102.6 μg ml^−1^ in compared to AA (7.21 μg ml^−1^). Moreover, antioxidant activity of bimetallic B_2_O_3_–ZnO NPs at concentrations 1000, 500, 250, 125 and 62.5 were 86.3, 69.3, 61.6, 55.4 and 35.3 μg ml^−1^, respectively.

Researchers showed that the combination of silver and zinc oxide, which forms plant-based nanomaterials, boosts their antioxidant capacity and their anti-proliferative activity eliminates free radicals.^103^ Therefore, bimetallic Ag/ZnO NPs produced *via* greener chemistry employing fenugreek plant had greater antioxidant capacity than monometallic silver and zinc oxide nanoparticles. Ag–ZnO NPs as an antioxidant agent may also be useful for treating liver and cancer diseases.^104^

Additionally, at a concentration of 500 μg ml^−1^, the biosynthesized bimetallic Ag–ZnO NPs from *Elephantopus scaber* L. demonstrated strong antioxidant activity of 62%. A silver–platinum bimetallic nano-alloy synthesized from *Vernonia mespilifolia* extract also showed potential antioxidant activity with an IC_50_ of 19.5 μg ml^−1^.^105^ Riaz *et al.*,^106^ reported that antioxidant activity was determined in the biosynthesized silver–nickel utilizing *Salvadora persica*, and scavenging activity was 70.5% at 1500 μg ml^−1^.

## Conclusion

In this study, bimetallic boron oxide–zinc oxide nanoparticles were successfully synthesized using gamma-rays in the presence of gum arabic. Analytical methods allowed for a thorough identification of the produced bimetallic NPs. When combined with the prepared gum arabic, the generated synthesized NPs exhibit consistent B_2_O_3_–ZnO NP surfaces with a transparent surface appearance. The corresponding brilliant spherical particles were discovered within the prepared gum arabic, and the surface characteristics and surface form of the generated synthesized NPs were examined. The synthesized nanocomposite, which appears as an illuminated NPs merged and capped with gum arabic, revealed that B_2_O_3_–ZnO NPs were fundamentally separated as spheroidal particles fused with one another across it. Through HR-TEM image, the synthetic bimetallic B_2_O_3_–ZnO NPs revealed that the particles were semi-spherical and attached with the stabilizing gum arabic, and their sizes ranged from 26.12 nm to 81.29 nm, with an average diameter of 53.97 ± 2.0 nm. Results revealed that bimetallic B_2_O_3_–ZnO NPs are safe in use according to cytotoxicity results on Vero and Wi38 normal cell lines. Additionally, bimetallic B_2_O_3_–ZnO NPs exhibited anticancer activity toward Caco 2 where IC_50_ was 80.1 μg ml^−1^. Furthermore, bimetallic B_2_O_3_–ZnO NPs showed promising antimicrobial activity against Gram-positive, and Gram-negative bacterial as well as unicellular fungi. Moreover, B_2_O_3_–ZnO NPs displayed antioxidant activity where IC_50_ was 102.6 μg ml^−1^.

## Conflicts of interest

The authors declare no conflict of interest.

## Supplementary Material

RA-013-D3RA03413E-s001

## References

[cit1] Davies J., Davies D. (2010). Microbiol. Mol. Biol. Rev..

[cit2] Sugden R., Kelly R., Davies S. (2016). Nat. Microbiol..

[cit3] Ferlay J., Colombet M., Soerjomataram I., Parkin D. M., Piñeros M., Znaor A., Bray F. (2021). Int. J. Cancer.

[cit4] Jamalipour Soufi G., Iravani P., Hekmatnia A., Mostafavi E., Khatami M., Iravani S. (2022). Comments Inorg. Chem..

[cit5] Duan X., Liao Y., Liu T., Yang H., Liu Y., Chen Y., Ullah R., Wu T. (2020). J. Photochem. Photobiol., B.

[cit6] Trayes K. P., Cokenakes S. E. (2021). Am. Fam. Physician.

[cit7] Ali O. M., Hasanin M. S., Suleiman W. B., Helal E. E.-H., Hashem A. H. (2022). Biomass Convers. Biorefin..

[cit8] Hashem A. H., Salem S. S. (2022). Biotechnol. J..

[cit9] Hashem A. H., Al Abboud M. A., Alawlaqi M. M., Abdelghany T. M., Hasanin M. (2022). Starch/Staerke.

[cit10] Ranjani S., Adnan M., Ruckmani K., Hemalatha S. (2020). Inorg. Nano-Met. Chem..

[cit11] Abu-Elghait M., Hasanin M., Hashem A. H., Salem S. S. (2021). Int. J. Biol. Macromol..

[cit12] Hashem A. H., Abdelaziz A. M., Askar A. A., Fouda H. M., Khalil A. M. A., Abd-Elsalam K. A., Khaleil M. M. (2021). J. Fungi.

[cit13] Dacrory S., Hashem A. H., Hasanin M. (2021). Environ. Nanotechnol., Monit. Manage..

[cit14] Elakraa A. A., Salem S. S., El-Sayyad G. S., Attia M. S. (2022). RSC Adv..

[cit15] Singh A. K., Xu Q. (2013). ChemCatChem.

[cit16] Seemala B., Cai C. M., Kumar R., Wyman C. E., Christopher P. (2018). ACS Sustainable Chem. Eng..

[cit17] Belenov S., Volochaev V., Pryadchenko V., Srabionyan V., Shemet D., Tabachkova N. Y., Guterman V. (2017). Nanotechnol. Russ..

[cit18] Zhang X., Yan S., Tyagi R., Surampalli R. (2011). Chemosphere.

[cit19] El-Batal A. I., Attia M. S., Nofel M. M., El-Sayyad G. S. (2019). J. Cluster Sci..

[cit20] Shah M., Fawcett D., Sharma S., Tripathy S. K., Poinern G. E. J. (2015). Materials.

[cit21] Sharma D., Kanchi S., Bisetty K. (2019). Arabian J. Chem..

[cit22] Hasanin M., Hashem A. H., Lashin I., Hassan S. A. M. (2023). Biomass Convers. Biorefin..

[cit23] Hashem A. H., Selim T. A., Alruhaili M. H., Selim S., Alkhalifah D. H. M., Al Jaouni S. K., Salem S. S. (2022). J. Funct. Biomater..

[cit24] Saied E., Salem S. S., Al-Askar A. A., Elkady F. M., Arishi A. A., Hashem A. H. (2022). Bioengineering.

[cit25] Albalawi M. A., Abdelaziz A. M., Attia M. S., Saied E., Elganzory H. H., Hashem A. H. (2022). Antioxidants.

[cit26] Hashem A. H., Saied E., Ali O. M., Selim S., Al Jaouni S. K., Elkady F. M., El-Sayyad G. S. (2023). Appl. Biochem. Biotechnol..

[cit27] Salem S. S., Ali O. M., Reyad A. M., Abd-Elsalam K. A., Hashem A. H. (2022). J. Fungi.

[cit28] Sivaselvam S., Selvakumar R., Viswanathan C., Ponpandian N. (2021). Chemosphere.

[cit29] El-Batal A. I., Nada H. G., El-Behery R. R., Gobara M., El-Sayyad G. S. (2020). RSC Adv..

[cit30] Fathy R. M., Mahfouz A. Y. (2021). J. Nanostruct. Chem..

[cit31] Saad A. M., El-Saadony M. T., El-Tahan A. M., Sayed S., Moustafa M. A., Taha A. E., Taha T. F., Ramadan M. M. (2021). Saudi J. Biol. Sci..

[cit32] El-Batal A. I., El-Sayyad G. S., Al-Hazmi N. E., Gobara M. (2019). J. Cluster Sci..

[cit33] Van de Loosdrecht A., Beelen R., Ossenkoppele G., Broekhoven M., Langenhuijsen M. (1994). J. Immunol. Methods.

[cit34] Khalil A., Abdelaziz A., Khaleil M., Hashem A. (2021). Lett. Appl. Microbiol..

[cit35] Clinical Laboratory Standards Institute (CLSI), 2002, M51-A2

[cit36] Shehabeldine A. M., Hashem A. H., Wassel A. R., Hasanin M. (2022). Appl. Biochem. Biotechnol..

[cit37] Hashem A. H., Hasanin M., Kamel S., Dacrory S. (2022). Colloids Surf., B.

[cit38] Hasanin M., Hashem A. H., El-Rashedy A. A., Kamel S. (2021). Cellulose.

[cit39] Valgas C., Souza S. M. D., Smânia E., Smânia A. (2007). Braz. J. Microbiol..

[cit40] Hashem A. H., Khalil A. M. A., Reyad A. M., Salem S. S. (2021). Biol. Trace Elem. Res..

[cit41] Hashem A. H., Shehabeldine A. M., Abdelaziz A. M., Amin B. H., Sharaf M. H. (2022). Appl. Biochem. Biotechnol..

[cit42] Bradford M. M. (1976). Anal. Biochem..

[cit43] de Menezes H. D., Tonani L., Bachmann L., Wainwright M., Braga G. Ú. L., von Zeska Kress M. R. (2016). J. Photochem. Photobiol., B.

[cit44] Paramanantham P., Antony A. P., Lal S. S., Sharan A., Syed A., Ahmed M., Alarfaj A. A., Busi S., Maaza M., Kaviyarasu K. (2018). Sci. Afr..

[cit45] Yildirim A., Mavi A., Kara A. (2000). J. Agric. Food Chem..

[cit46] El-Baz A. F., El-Batal A. I., Abomosalam F. M., Tayel A. A., Shetaia Y. M., Yang S. T. (2016). J. Basic Microbiol..

[cit47] El-Batal A. I., El-Sayyad G. S., El-Ghamry A., Agaypi K. M., Elsayed M. A., Gobara M. (2017). J. Photochem. Photobiol., B.

[cit48] El-Batal A. I., Mosallam F. M., El-Sayyad G. S. (2018). J. Cluster Sci..

[cit49] Borokhov O. and Schubert D., Antimicrobial Properties of Boron Derivatives, in ASC Symposium Series, Oxford University Press, 2007, pp. 412–435

[cit50] Kolya H., Pal S., Pandey A., Tripathy T. (2015). Eur. Polym. J..

[cit51] Yuzheng W., Xiangxin X., He Y. (2014). Chin. J. Chem. Eng..

[cit52] Hareesh K., Sanjeev G., Pandey A., Rao V. (2013). Iran. Polym. J..

[cit53] El-Batal A. I., El-Sayyad G. S., El-Ghamery A., Gobara M. (2017). J. Cluster Sci..

[cit54] Barabadi H., Honary S., Ebrahimi P., Alizadeh A., Naghibi F., Saravanan M. (2019). Inorg. Nano-Met. Chem..

[cit55] El-Batal A. I., El-Sayyad G. S., Al-Hazmi N. E., Gobara M. (2019). J. Cluster Sci..

[cit56] El-Batal A. I., El-Sayyad G. S., Mosallam F. M., Fathy R. M. (2020). J. Cluster Sci..

[cit57] El-Batal A. I., Attia M. S., Nofel M. M., El-Sayyad G. S. (2019). J. Cluster Sci..

[cit58] Attia M. S., El-Sayyad G. S., Saleh S. S., Balabel N. M., El-Batal A. I. (2019). J. Cluster Sci..

[cit59] Sui M., Kunwar S., Pandey P., Lee J. (2019). Sci. Rep..

[cit60] Kong H., Yang J., Zhang Y., Fang Y., Nishinari K., Phillips G. O. (2014). Int. J. Biol. Macromol..

[cit61] Wu C.-C., Chen D.-H. (2010). Gold Bull..

[cit62] Fouda A., Salem S. S., Wassel A. R., Hamza M. F., Shaheen T. I. (2020). Heliyon.

[cit63] Munir R., Ali K., Naqvi S. A. Z., Maqsood M. A., Bashir M. Z., Noreen S. (2023). Sep. Purif. Technol..

[cit64] Kelly K. L., Coronado E., Zhao L. L., Schatz G. C. (2003). J. Phys. Chem. B.

[cit65] Prasad K. S., Selvaraj K. (2014). Biol. Trace Elem. Res..

[cit66] Castro-Longoria E., Vilchis-Nestor A. R., Avalos-Borja M. (2011). Colloids Surf., B.

[cit67] Lawrie A., Albanyan A., Cardigan R., Mackie I., Harrison P. (2009). Vox Sang..

[cit68] Monika P., Chandraprabha M., Hari Krishna R., Vittal M., Likhitha C., Pooja N., Chaudhary V. (2022). Biotechnol. Genet. Eng. Rev..

[cit69] El-Batal A. I., Abd Elkodous M., El-Sayyad G. S., Al-Hazmi N. E., Gobara M., Baraka A. (2020). Int. J. Biol. Macromol..

[cit70] Nissen M., Förster R., Wieduwilt T., Lorenz A., Jiang S., Hauswald W., Schmidt M. A. (2022). Small.

[cit71] Souza T. G. F. (2016). et al.. J. Phys.: Conf. Ser..

[cit72] Mohsin M., Jawad M., Yameen M. A., Waseem A., Shah S. H., Shaikh A. J. (2020). Plasmonics.

[cit73] Hasija V., Sharma K., Kumar V., Sharma S., Sharma V. (2018). Vacuum.

[cit74] Bigdeli F., Morsali A. (2010). Mater. Lett..

[cit75] Acharya S., Karmakar S., Dooley K. M. (2012). J. Propul. Power.

[cit76] Poyraz S., Cerkez I., Huang T. S., Liu Z., Kang L., Luo J., Zhang X. (2014). ACS Appl. Mater. Interfaces.

[cit77] Belavi P., Chavan G., Naik L., Somashekar R., Kotnala R. (2012). Mater. Chem. Phys..

[cit78] Pal K., Elkodous M. A., Mohan M. M. (2018). J. Mater. Sci.: Mater. Electron..

[cit79] Ioset J.-R. , Brun R., Wenzler T., Kaiser M. and Yardley V., A Training Manual for Screening in Neglected Diseases, 2009

[cit80] Cao Y., Dhahad H. A., El-Shorbagy M. A., Alijani H. Q., Zakeri M., Heydari A., Bahonar E., Slouf M., Khatami M., Naderifar M., Iravani S., Khatami S., Dehkordi F. F. (2021). Sci. Rep..

[cit81] Hashem A. H., El-Sayyad G. S. (2023). Biomass Convers. Biorefin..

[cit82] Makada H., Habib S., Singh M. (2023). Sci. Afr..

[cit83] Elsayed K. A., Alomari M., Drmosh Q. A., Alheshibri M., Al Baroot A., Kayed T. S., Manda A. A., Al-Alotaibi A. L. (2022). Alexandria Eng. J..

[cit84] Nieto-Argüello A., Medina-Cruz D., Pérez-Ramírez Y. S., Pérez-García S. A., Velasco-Soto M. A., Jafari Z., De Leon I., González M. U., Huttel Y., Martínez L. (2022). Nanomaterials.

[cit85] Nalvolthula R., Merugu R., Koyyati R., Rudra M. P. (2023). Int. J. ChemTech Res..

[cit86] Alafaleq N. O., Zughaibi T. A., Jabir N. R., Khan A. U., Khan M. S., Tabrez S. (2023). Nanomaterials.

[cit87] Kim U., Kim C.-Y., Lee J. M., Oh H., Ryu B., Kim J., Park J.-H. (2020). Pathol. Oncol. Res..

[cit88] López Grueso M. J., Tarradas Valero R. M., Carmona-Hidalgo B., Lagal Ruiz D. J., Peinado J., McDonagh B., Requejo Aguilar R., Bárcena Ruiz J. A., Padilla Peña C. A. (2019). Antioxidants.

[cit89] Rodriguez J. A., Goodman D. W. (1992). Science.

[cit90] Stamenkovic V. R., Fowler B., Mun B. S., Wang G., Ross P. N., Lucas C. A., Markovic N. M. (2007). Science.

[cit91] Beyth N., Houri-Haddad Y., Domb A., Khan W., Hazan R. (2015). Evidence-Based Complementary Altern. Med..

[cit92] Ramakritinan C., Kaarunya E., Shankar S., Kumaraguru A. (2013). Solid State Phenom..

[cit93] Malapermal V., Mbatha J. N., Gengan R. M., Anand K. (2015). Adv. Mater. Lett..

[cit94] Khatak S., Wadhwa N., Jain P. (2021). Biosci., Biotechnol. Res. Asia.

[cit95] Hu M., Li C., Li X., Zhou M., Sun J., Sheng F., Shi S., Lu L. (2018). Artif. Cells, Nanomed., Biotechnol..

[cit96] Merugu R., Gothalwal R., Kaushik Deshpande P., De Mandal S., Padala G., Latha Chitturi K. (2021). Mater. Today: Proc..

[cit97] Yin H., Xu L., Porter N. A. (2011). Chem. Rev..

[cit98] Premanathan M., Karthikeyan K., Jeyasubramanian K., Manivannan G. (2011). Nanomedicine.

[cit99] Dutta R. K., Nenavathu B. P., Gangishetty M. K., Reddy A. V. (2012). Colloids Surf., B.

[cit100] Phaniendra A., Jestadi D. B., Periyasamy L. (2015). Indian J. Clin. Biochem..

[cit101] Li Y., Li X., Wong Y.-S., Chen T., Zhang H., Liu C., Zheng W. (2011). Biomaterials.

[cit102] Tinggi U. (2008). Environ. Health Prev. Med..

[cit103] Sánchez-López E., Gomes D., Esteruelas G., Bonilla L., Lopez-Machado A. L., Galindo R., Cano A., Espina M., Ettcheto M., Camins A. (2020). Nanomaterials.

[cit104] Noohpisheh Z., Amiri H., Farhadi S., Mohammadi-gholami A. (2020). Spectrochim. Acta, Part A.

[cit105] Unuofin J. O., Oladipo A. O., Msagati T. A., Lebelo S. L., Meddows-Taylor S., More G. K. (2020). Arabian
J. Chem..

[cit106] Riaz T., Mughal P., Shahzadi T., Shahid S., Abbasi M. A. (2020). Mater. Res. Express.

