# Correction: Unveiling anticancer, antimicrobial, and antioxidant activities of novel synthesized bimetallic boron oxide–zinc oxide nanoparticles

**DOI:** 10.1039/d3ra90066e

**Published:** 2023-07-27

**Authors:** Amr H. Hashem, Samar H. Rizk, Mostafa A. Abdel-Maksoud, Wahidah H. Al-Qahtani, Hamada AbdElgawad, Gharieb S. El-Sayyad

**Affiliations:** a Botany and Microbiology Department, Faculty of Science, Al-Azhar University Nasr City Cairo 11884 Egypt amr.hosny86@azhar.edu.eg; b Department of Biochemistry, Faculty of Pharmacy, Ahram Canadian University Sixth of October City Giza Egypt; c Department of Biochemistry, Faculty of Pharmacy, Galala University New Galala City Suez Egypt; d Botany and Microbiology Department, College of Science, King Saud University P.O. Box 2455 Riyadh 11451 Saudi Arabia; e Department of Food Sciences & Nutrition, College of Food and Agricultural Sciences, King Saud University P.O. Box 270677 Riyadh 11352 Saudi Arabia; f Integrated Molecular Plant Physiology Research, Department of Biology, University of Antwerp 2020 Antwerp Belgium; g Microbiology and Immunology Department, Faculty of Pharmacy, Ahram Canadian University Sixth of October City Giza Egypt; h Microbiology and Immunology Department, Faculty of Pharmacy, Galala University New Galala City Suez Egypt Gharieb.Elsayyad@gu.edu.eg; i Drug Microbiology Lab, Drug Radiation Research Department, National Center for Radiation Research and Technology (NCRRT), Egyptian Atomic Energy Authority (EAEA) Cairo Egypt

## Abstract

Correction for ‘Unveiling anticancer, antimicrobial, and antioxidant activities of novel synthesized bimetallic boron oxide–zinc oxide nanoparticles’ by Amr H. Hashem *et al.*, *RSC Adv.*, 2023, **13**, 20856–20867, https://doi.org/10.1039/D3RA03413E.

The authors regret that one of the affiliations (affiliation *f*) was incorrectly shown in the original manuscript. The corrected list of affiliations is as shown above.

The Royal Society of Chemistry apologises for these errors and any consequent inconvenience to authors and readers.

## Supplementary Material

