# Supplementary material for: Unveiling anticancer, antimicrobial, and antioxidant activities of novel synthesized bimetallic boron oxide–zinc oxide nanoparticles
Source: RSC Adv. 2023 Jul 12;13(30):20856–67. doi: 10.1039/d3ra03413e (PMC10336335; doi:10.1039/d3ra03413e)
Supplement: RA-013-D3RA03413E-s001 [file RA-013-D3RA03413E-s001.pdf]

Electronic Supplementary Material (ESI) for RSC Advances.
This journal is © The Royal Society of Chemistry 2023

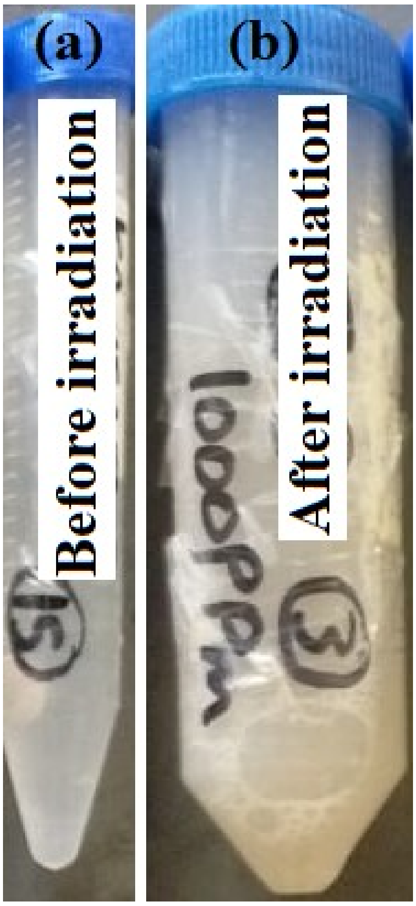

Figure S1: The change in color before and after gamma irradiation where (a), the faint white color of the prepared synthetic solution, and (b) the deep off-white color of the synthesized bimetallic B_2O_3-ZnO NPs.
